# Synthetic biotechnology for C1 bio-refinery

**DOI:** 10.1016/j.synbio.2024.09.009

**Published:** 2024-09-23

**Authors:** Yongjin Zhou, Benjamin Woolston

**Affiliations:** Dalian Institute of Chemical Physics, Chinese Academy of Sciences, China; Northeastern University, USA

The transition to a sustainable of bioeconomy requires the expanding of feedstocks for bio-manufacturing. Single Carbon (C1) feedstocks, including CO_2_, CO, CH_4_, formate and methanol, can serve as ideal raw materials for production of chemicals and biofuels toward the goal of carbon neutrality. Synthetic biology provides advanced genetic engineering techniques and tools for construction of efficient bioprocesses for bioconversion of C1 feedstocks. Synthetic and Systems Biotechnology organized a special issue about current progress in this area, comprising four reviews and six original research articles.

## 1 Synthetic biology tools for C1 microbes

Synthetic biology tools for C1 microbes would accelerate the engineering microbial cell factories, but most of these organisms are reluctant to be engineered. Methanotrophic bacteria are widely used industrially for the bioconversion of methane-rich natural gas toward valuable products. However, their limited genetic tools hinders metabolic engineering of these biocatalysts. Bhat et al. constructed a broad-host-range Anderson promoter series and particulate methane monooxygenase promoter variants, which advanced the genetic engineering toolbox for the model methanotroph *Methylococcus capsulatus* (https://doi.org/10.1016/j.synbio.2024.02.003). The methylotrophic yeast *Komagataella phaffii* (syn. *Pichia pastoris*) has been widely used for protein production for decades and shows great potential for producing small molecule by using methanol as a carbon source. An improved CRISPR based genome engineering with recyclable selective marker was developed for multiple-step metabolic engineering, which was applied for the inositol production from methanol in *K*. *phaffii* (https://doi.org/10.1016/j.synbio.2023.06.003). An improved CRISPR interference (CRISPRi) system was developed by fusing dCas9 with endogenous transcriptional repressor domains, which enabled multiplexed gene repression in *K*. *phaffii* (https://doi.org/10.1016/j.synbio.2023.06.008). These genetic engineering tools should accelerate the engineering efficient microbes for C1 bio-refinery.

## 2 Engineering efficient enzymatic catalysis

Certain key enzymes in C1 metabolism are inefficient, bottlenecking growth and production. The methanol bioconversion in methylotrophic bacteria is limited by the poor catalytic properties of NAD^+^-dependent methanol dehydrogenase (Mdh) that oxidizes methanol to formaldehyde. Qian et al. engineered the neutrophilic and mesophilic NAD + -dependent Mdh from *Bacillus stearothermophilus* by directed evolution for enhancing the catalytic activity by 6.5-fold (https://doi.org/10.1016/j.synbio.2023.05.004). Ribulose-1,5-bisphosphate carboxylase/oxygenase (Rubisco) is the key enzyme that mediates CO_2_ fixation. However, its low activity greatly hinders carbon fixation efficiency. A review by Zhao et al. comprehensively discussed the reconstruction of Rubisco in microorganisms for CO_2_ utilization (https://doi.org/10.1016/j.synbio.2023.12.006). Another review by Qiao et al. comprehensively overviewed the recent advances in the construction of in *vitro* multi-enzyme cascades and whole cells for C1 biotransformation as well as the challenges and possible solutions in upscaling multi-enzyme cascades (https://doi.org/10.1016/j.synbio.2023.08.008).

## 3 Cell factory and bioprocess for C1 conversion

Cordycepin is a potential alternative to the disputed herbicide glyphosate. Tan et al. engineered a *K*. *phaffii* cell factory for cordycepin production from methanol at g/L titers, and analyzed the engineered cell with transcriptome analysis (https://doi.org/10.1016/j.synbio.2023.03.003). C1 carbons can be also used as a co-substrate to improve the bioproduction with other feedstocks such as sugar. Zhao et al. used formate as a supplementary substrate to improve the sugar metabolism and solvent production in *Clostridium beijerinckii*, which improved ABE (acetone-butanol-ethanol) production by 50 % (https://doi.org/10.1016/j.synbio.2023.01.005). Though the C1 biotransformation is developing rapidly, there are many obstacles toward industrial applications. Two reviews by Barmschaubl et al. and Sarwar et al. comprehensively discussed the challenges in engineering efficient single carbon metabolism (https://doi.org/10.1016/j.synbio.2024.03.003) as well as methanol metabolism (https://doi.org/10.1016/j.synbio.2023.06.001) in microbes.

Collectively, the papers in this issues showcase the application of synthetic and systems biology to enhancing C1 bio-conversion across a wide range of platform organisms and substrates toward the goal of carbon neutral bio-manufacturing. We thank all contributing authors for making this special issue on “Synthetic biotechnology for C1 bio-refinery” possible, and also the reviewers for their constructive comments to improve the manuscripts. We hope that readers find the articles interesting and inspiring to their own research.

